# A Super-Microsurgery Training Model: The Mouse Caudal Artery Anastomosis Model

**DOI:** 10.3389/fsurg.2022.841302

**Published:** 2022-04-07

**Authors:** Xue-Qiang Wu, Hui-Ren Liu, Zhan-Yong Yu, Yan Wang, Ru-Tao Sun, Li Wang, Yuan Gao

**Affiliations:** ^1^Department of Handsurgery, Tangshan Second Hospital, Tangshan, China; ^2^Institute of Trauma Surgery, Tangshan Second Hospital, Tangshan, China

**Keywords:** vascular anastomosis, microsurgery, mouse, mouse caudal artery, model

## Abstract

**Objective:**

To establish a simple and practical model for super-microsurgery training using the middle caudal arteries of Kunming mice.

**Methods:**

A ⊔-shaped incision was made approximately 1 cm from the root of the tail in the mouse, and the skin, together with the subcutaneous tissue, was turned up into a rectangular shape to the opposite side with exposure of the mouse middle caudal artery and the accompanying veins. The artery was freed for approximately 1 cm in length. The middle caudal artery was cut transversely at the site, and then the severed middle caudal artery was anastomosed end-to-end using 12-0 microsutures in the order of 6, 12, 3, and 9 o'clock with four stitches.

**Results:**

The mouse caudal artery had a constant anatomical location accompanied by a vein. The immediate postoperative patency after vascular anastomosis was 100% (15/15) in all mouse models, the postoperative patency was 100% (5/5), 80% (4/5), and 75% (3/4) at 24 h, 3 days, and 1 week postoperatively, respectively. The outer diameter of the mouse middle caudal artery was 0.2 ~ 0.3 (0.22 ± 0.03) mm. The vascular anastomosis time was 6.5 ~ 15 (11.0 ± 2.5) min.

**Conclusion:**

The mouse middle caudal artery was superficially located and anatomically constant, making it easy to be located and exposed. The small size of the opening made it suitable for establishing a useful model for training in super-microsurgery vascular anastomoses.

## Introduction

With the development of microsurgery technology, restorative surgery has become more delicate. New technical operations, such as fingertip replantation, free grafting of perforator flaps, and lymphatic vessel anastomosis, have emerged, requiring advanced microsurgical techniques. In 1998, Koshima et al. (1) called the perforator flap technique “super-microsurgery” for the first time. And in 2010, Koshima (2) proposed the definition “Super-microsurgery is a technique of microneurovascular anastomosis for smaller vessels and single nerve fascicle, and also microneurovascular dissection for these small vessels less than 0.3 to 0.8 mm.” Since then, super-microsurgery techniques have become increasingly applied across clinical practice, requiring clinicians to have high-level skills in hand–eye coordination under the microscope, patience during microsurgical operations, and control and balance while using microscopic instruments. To achieve clinical proficiency, the usual systematic, specialized, and microsurgical training is required. Commonly used training models include materials such as rubber gloves, silicone tubes, fresh or frozen chicken wings, or chicken leg veins (3–6). However, the models that are closer in similarity to actual clinical operations comprise animals, such as rabbits and rats (7, 8). The rat caudal artery anastomosis is more frequently adopted (9), but the vessel diameter is relatively large and differs from that of super-microsurgery anastomosis. To explore the vascular anastomosis model, which is more difficult yet more suitable for super-microsurgery training, the smaller size mice were selected to reveal and anastomose the middle caudal artery; this presented the possibility of establishing a super-microsurgery training model using the mouse middle caudal artery.

## Materials and Methods

### Materials

Fifteen Kunming male or female mice (Vital River Laboratory Animal Technology Co., Ltd., Beijing, China), specific-pathogen-free (SPF) grade, with the body weight of 37.1 ~ 48.5 (42.3 ± 4.3) g were used in this study.

One operating microscope (Leica M500-N, Germany), a set of surgical microscopy instruments (Cheng-He, Ningbo Cheng-He Microsurgical Instruments Factory, Ningbo, China), several conventional surgical instruments, one blunt-ended flushing needle (4#), angiometric calipers, a timer, 12-0 non-invasive sutures (Crownjun, Japan), 10% chloral hydrate, and heparin sodium isotonic saline (25 U/ml) were the instruments and agents used in this study.

### Methods

**Figure 1:**
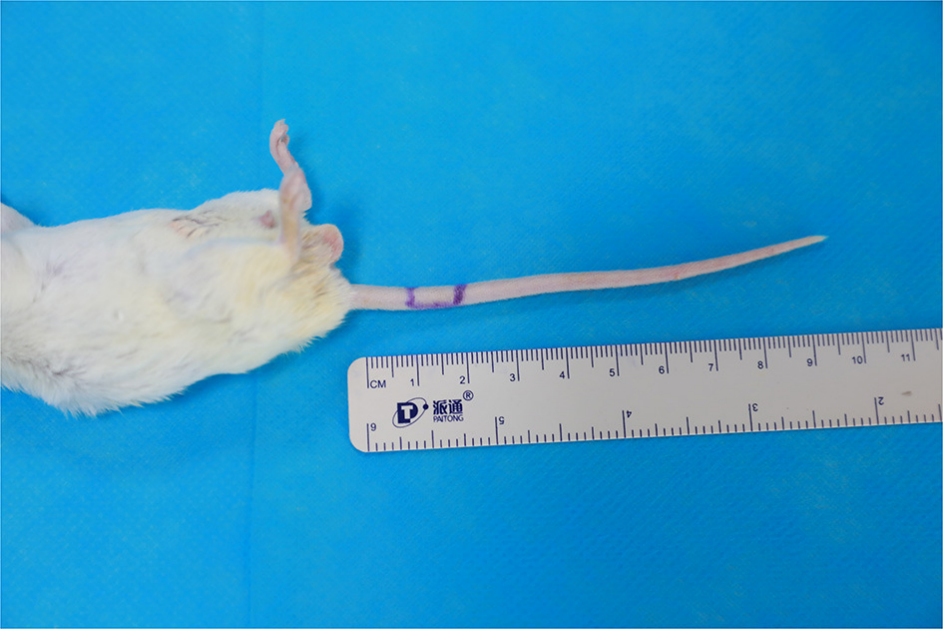
A ⊔-shaped incision was made one centimeter from the root of the mouse tail.

**Figure 2:**
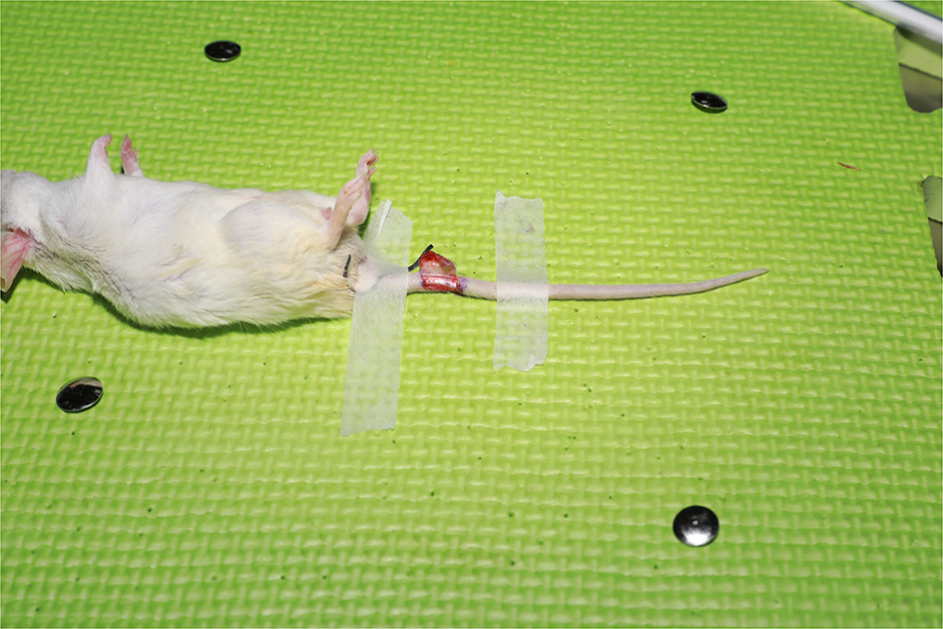
The skin was turned up into a rectangular shape to the opposite side with exposure of the mouse middle caudal artery and the accompanying veins.

**Figure 3:**
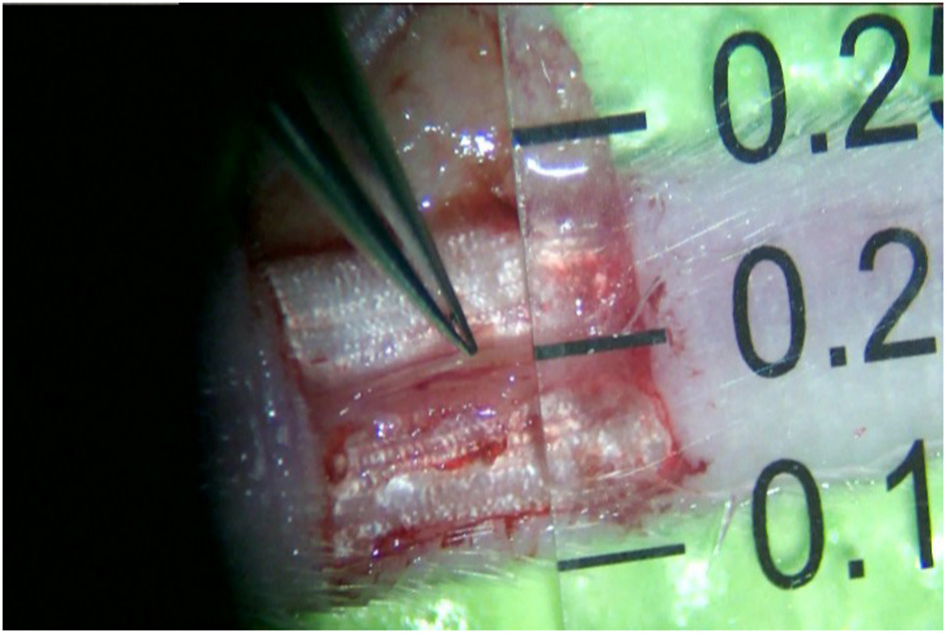
The mouse middle caudal artery was exposed with the measurement of the diameter.

**Figure 4:**
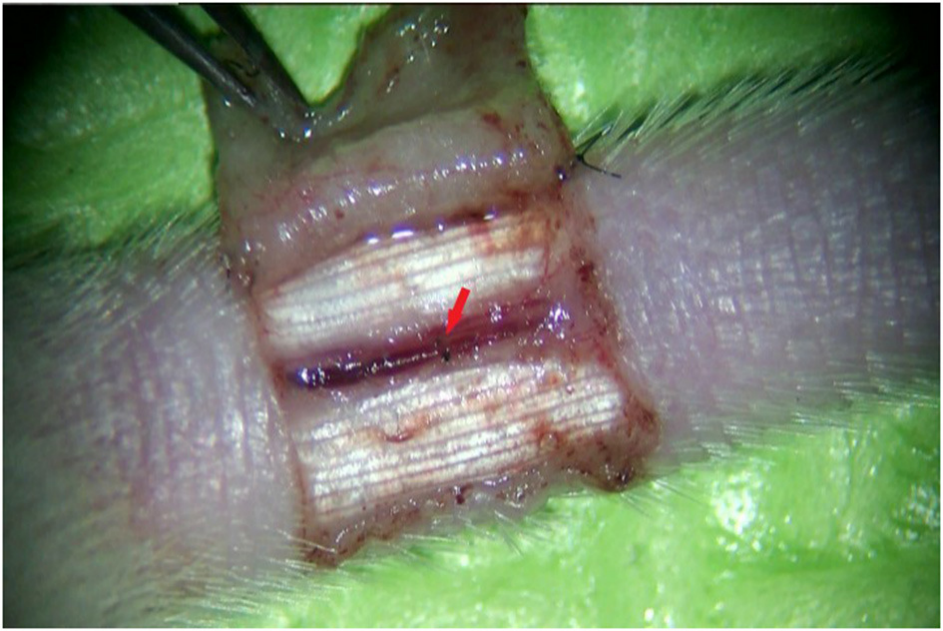
The successfully anastomosed mouse middle caudal artery. The red arrow shows the anastomosis site.

All mice were housed in a single cage with no limitation on water and fasted for 12 h before surgery. The mice were anesthetized intraperitoneally with 10% chloral hydrate at a dose of 0.2 ml/50 g of their body weight. A relatively aseptic technique was adopted. The mice were fixed abdominally upward on a special animal spreader to ensure that breathing was neither shallow and fast nor deep and slow. A rubber strip was used to achieve hemostasis at the root of the mouse tail. The skin and subcutaneous tissues were incised after iodophor disinfection, and a ⊔-shaped incision was made ~1 cm from the root of the mouse tail (Figure 1). The skin, together with the subcutaneous tissue, was turned up into a rectangular shape to the opposite side (Figure 2). The vascular sheath encircling the mouse caudal artery was sharply separated under a 13X microscope, the middle caudal artery was exposed by cutting with microscopic scissors, and the diameter of the vessel was measured using angiometric calipers (Figure 3). The middle caudal artery was located ventrally in the mouse tail and was accompanied by a vein. The artery was freed for approximately 1 cm in length. In cases of obvious arterial spasm after freeing, a drop of 2% lidocaine hydrochloride solution was applied locally. After the spasm was relieved, the mouse middle caudal artery was transected at the site, and heparin saline (100 U/ml) was used to flush out the residual blood in the lumen and remove the outer membrane tissue and foreign bodies around the dissected end of the vessel. After that, 12-0 microscopic sutures (Crownjun, Japan), 14 cm curved needle holder, 14 cm curved microsurgery scissors, and 15 cm microsurgery forceps with the tip of 0.15 mm were used. The first stitch fit the back wall (6 o'clock), with at least three knots for each stitch. Then the anterior and lateral walls were anastomosed end-to-end in the order of 12, 3 and 9 o'clock (Figure 4). The distance between the sutures was one to two times the thickness of the vessel wall.

With the completion of the intraoperative vascular anastomosis, the rubber band at the root of the mouse tail was released after conduction of complete hemostasis. The patency of the vessels was immediately observed using the strangulation method and observations were recorded. The wound was closed with interrupted 6-0 nylon monofilament sutures. All surgical operations were performed under a surgical microscope with 13-fold magnification by the same operator and assistant, and no anticoagulants were applied postoperatively.

The outer diameter of the vessel was measured intraoperatively, the vessel anastomosis time (time from cutting the vessel to completion of suturing) was recorded, and the immediate patency of the anastomosis was observed. Following these procedures, 15 mice were randomly divided into three groups: the 24-h group (group A), the three-day group (group B), and the one-week group (group C). The anastomosed middle caudal artery was re-anesthetized at 24 h, 3 days, and 1 week after surgery, and the anastomosed artery was cut at approximately 0.5 cm distal to the vascular anastomosis to observe the bleeding at the proximal end of the vessel in order to determine whether the vessel was patent. After observation, the mice were executed by the medullary dissection method.

## Results

All 15 mice that underwent operation were awakened postoperatively; three of them were slightly less vigorous and one from group C died on the fourth postoperative day. The immediate patency after vascular anastomosis was 100% (15/15) in all mouse models, and the postoperative patency was 100% (5/5) in group A, 80% (4/5) in group B, and 75% (3/4) in group C, respectively. The outer diameter of the mouse middle caudal artery was 0.2 ~ 0.3 (0.22 ± 0.03) mm. The vascular anastomosis time was 6.5 ~ 15 (11.0 ± 2.5) min.

## Discussion

There are three main types of well-established models used for microsurgery training: non-biological material models, such as latex gloves and glue tubes (3, 4); non-living tissue models, such as chicken wings, avulsed skin, and placenta (5, 10, 11); and living animal models, such as rats (8, 9). The non-biological material models are low cost and are often used by surgeons for more preliminary training, such as familiarization with operating microsurgical instruments and knot tying. After training with the non-biological materials, non-living tissue models can be used to practice microsurgery. Materials like chicken wings and legs (5, 6), commonly used for microsurgery training, can be purchased from the market and are inexpensive, convenient, and easily available. However, there is a disadvantage to utilizing this model in that the hemodynamic patency of the anastomosis cannot be confirmed. In addition, the adoption of discarded materials from surgical procedures, such as avulsed skin, placenta, or skin tissue removed during abdominoplasty, has been reported for use in training (10–12). This application is limited, however, due to ethical limitations and the fact that the materials are not easily or consistently available. Although non-biological materials and non-living tissue models cannot replace the living animal models, they are essential for training surgeons in hand–eye coordination under the microscope as a supplement and adjuvant to animal models. Among the animal models, the caudal and carotid arteries in rats are thicker in diameter and are easily separated, making them more often adopted in training (9, 13).

In 1997, at the first international course on Penetrating and Arterialized Flaps in Ghent, Belgium, Prof. Koshima reported the application of penetrating vessels <0.8 mm in diameter as the basis for cutting penetrating flaps (14), and the safe anastomosis of these vessels was conducted to reconstruct soft tissue defects in different parts of the body. In 1998, Koshima et al. (1) first referred to this technique as “super-microsurgery” when describing the paramedian penetrating flap. In 2010, Koshima (2) defined the technique of anastomosis and separation of small vessels with a diameter of 0.3 to 0.8 mm as the category of super-microsurgery. This requires having microsurgeons with fine operating techniques and excellent hand–eye coordination. However, with the progressive training model described above, only general microsurgery skills can be acquired for anastomosis of thicker vessels with a diameter over 1.0 mm, but not fully competent for anastomosis of vessels <0.8 mm in diameter.

In super-microsurgery training, the most basic models include practice cards containing silicone tubes with a diameter of <0.8 mm, which can be used to practice anastomosis of vessels in super-microsurgery (15). Crownjun Co. has a 0.25 cm vascular model, but it is very expensive and not suitable for daily massive training. In the chicken thigh model, the branches of the sciatic artery and vein are between 0.3 and 0.5 mm in diameter, allowing manipulation on biological sub-millimeter vessels (6). Although the chicken thigh is a non-living biological model, trainees can receive nearly the same tactile feedback as they would if operating on a patient. Moreover, the cost is low and pre- or postoperative care is not needed. *In vivo* biological models are mainly rats. Ozkan et al. (16) reported that the diameter of the vascular pedicle of the superficial inferior epigastric artery (SIEA) perforator flap in rats is similar to the diameter of human perforator vessels, lymphatic vessels, and finger arteries, and it is a good animal model for training in super-microsurgery techniques. Mofikoya et al. (17) also measured the flap model, and further demonstrated the feasibility with the adoption of this vascular pedicle to cut a free flap, and it was confirmed that this flap might be suitable for super-microsurgery training.

The diameter of the anastomosed blood vessel in super-microsurgery is 0.30 ~ 0.8 mm, while the diameter of a mouse middle caudal artery is 0.20 ~ 0.30 mm, which can fully meet the requirements of super-microsurgery for vascular anastomosis. Compared with the rat SIEA (18) perforating branch (0.4 mm), the mouse caudal artery is thinner, which requires advanced microsurgery techniques and presents more difficulty in training. Moreover, the mouse caudal artery is in a single plane and superficially located, making it easy to operate on and more suitable for simple vascular anastomosis training. The blood vessels in the mouse tail are easier to conduct hemostasis, which can prevent death in mice due to hemorrhage. Advantages to using mice are they are small in size, easy to control regarding the amount of anesthetic dose, relatively inexpensive, and easy to feed. The tail anastomosis of mice belongs to *in vivo* anastomosis, and it can be observed whether the blood vessels are patent after anastomosis, to test the quality of anastomosis. The separable length of the rat SIEA is only approximately 12–14 mm (18), while the longer length of the mouse caudal artery allows for multiple anesthesia and multiple anastomoses.

At 24 h, 3 days, and 1 week after surgery, due to local tissue edema and increased vascular fragility, the strangulation method showed a tendency to damage the vessels and tear the anastomosis in the pre-test, so we did not use the traditional strangulation method for the observation of patency. In contrast, the middle caudal artery was cut at the site approximately 0.5 cm distal to the anastomosis and the patency of the vessel was ascertained by observing the bleeding at the proximal end. Active bleeding at the proximal end after cutting was considered to be a patency of the anastomosis.

The immediate patency after vascular anastomosis and the patency after 24 h were both 100%, demonstrating the feasibility of vascular anastomosis in the mouse caudal artery despite the small diameter. The patency of the anastomosed vessels was reduced at 3 days and 1 week after surgery, and it was considered to be due to the low blood pressure and slow blood flow in the mouse middle caudal artery, which had a higher risk of thrombus after surgery. Secondly, non-administration of anticoagulants might also be a factor in the decreased patency. In addition, the maximum magnification of the surgical microscope in our laboratory was <14X, while the vascular anastomosis used by Chen et al. was conducted under a 21.3X microscope (6). Whether conducting vascular anastomosis under a clearer and larger magnification microscope could improve the long-term vascular patency is a question that we should focus on in the future. Although it was difficult to draw clinical conclusions from experimental studies, the model might provide a training tool for obtaining more refined super-microsurgery techniques. Finally, suture type might also affect the success rate of vascular anastomosis. A 12-0, 50 μm microsuture was used for the mouse caudal artery anastomosis, which could ensure competent training for 0.2 mm microvascular anastomosis. A13-0, 30 μm microscopic suture was reported by Yamamoto et al. (19), which was considered more suitable for the anastomosis of tiny vessels and lymphatic vessels of 0.3 mm in diameter and below and was essential for the development of super-microsurgery.

The limitation in the present study was that only the feasibility of super-microsurgery vascular anastomosis in the mouse middle caudal artery was confirmed through experiments; no detailed anatomical study of the mouse tail had been performed, nor had a replantation of the mouse tail been attempted.

## Data Availability Statement

The original contributions presented in the study are included in the article/supplementary material, further inquiries can be directed to the corresponding author/s.

## Ethics Statement

The animal study was reviewed and approved by Ethics Committee of Tangshan Second Hospital (TSEY-LL-2021063).

## Author Contributions

X-QW and H-RL: conception, design of the research, analysis, and interpretation of the data. X-QW, Z-YY, YW, and YG: acquisition of data. X-QW: statistical analysis and writing of the manuscript. X-QW, Z-YY, YW, R-TS, LW, and H-RL: critical revision of the manuscript for intellectual content. All authors read and approved the final draft.

## Conflict of Interest

The authors declare that the research was conducted in the absence of any commercial or financial relationships that could be construed as a potential conflict of interest.

## Publisher's Note

All claims expressed in this article are solely those of the authors and do not necessarily represent those of their affiliated organizations, or those of the publisher, the editors and the reviewers. Any product that may be evaluated in this article, or claim that may be made by its manufacturer, is not guaranteed or endorsed by the publisher.
